# Transition for adolescents with learning disabilities and an immunodeficiency

**DOI:** 10.3389/fimmu.2023.1211872

**Published:** 2023-09-13

**Authors:** Eliška Alderson, Sarah Lally, Mari Campbell

**Affiliations:** ^1^ Department of Clinical Immunology, Royal Free Hospital, London, United Kingdom; ^2^ Acute Liaison – Learning Disabilities, Royal Free Hospital, London, United Kingdom; ^3^ University College London (UCL) Institute of Immunity & Transplantation, London, United Kingdom

**Keywords:** transition, Immunodeficiency, learning disability, mental health, adolescent

## Abstract

Many adolescents with immunodeficiency are diagnosed with a comorbid learning disability. The process of transition from paediatric to adult healthcare for these individuals occurs with a range of additional challenges. Due to the lack research available on immunodeficiency specifically, this article addresses a number of recommendations from the research undertaken with individuals with other chronic health conditions and learning disability. The research suggests that for individuals with learning disabilities autonomy and independence needs to be acknowledged despite their perceived need for increased input from parents and medical professionals. Instead, medical professionals could prioritise their relationship with the adolescent patient by ensuring communication needs are met and that a sense of continuity between paediatric and adult services is maintained. Families can be supported through psychological interventions which provide skills to allow family members to empower their young adult with a learning disability. Specific tools to help the transition process run more smoothly are also recommended and have proven to be effective in other parts of the world.

## Introduction

Immunodeficiency is a general term used to describe a number of different disorders that result from an impairment of the immune system. Immunodeficiencies typically lead to increased susceptibility to infection but can also be associated with other complications. Immunodeficiencies may be broadly categorised into primary immunodeficiency (PID) or secondary immunodeficiency. Primary immunodeficiencies encompass a large number of genetic disorders which can also be termed as inborn errors of immunity whereas secondary immunodeficiencies often occur as a result of a medication or another disorder. In this paper, immunodeficiency will refer to primary immunodeficiency of inborn errors of immunity. Due to improvements in the identification and treatment of immunodeficiencies, patients are living longer, and more are requiring referral to adult services. Adolescents that live with deficits to their immune system, may also be diagnosed with learning disabilities which can greatly affect their quality of life. Cognitive and neurological difficulties are also specifically noted for some specific conditions, adenosine deaminase-deficient severe combined immunodeficiency (ADA-SCID) for example (1). These neurological manifestations in ADA-SCID are caused by variants to the ADA gene that result in accumulation of toxic metabolites in the brain particularly the thalamus and basal ganglia, leading to neurological function abnormalities such as motor abnormalities and learning disability (1). They may co-occur with the condition or result from treatment or the lack of it. Neurological symptoms can range from mild delay in cognition or language to more severe disabilities (2). Research has shown that different types of immunodeficiencies may present with higher rates of comorbid learning or intellectual disabilities, such as combined immunodeficiencies (3) and severe congenital immunodeficiencies (4). Research on children with severe congenital immunodeficiencies found that around 19% of their patient cohort could be classified as having a learning or intellectual disability (with an IQ < 70) (4). DiGeorge syndrome (22q11.2 deletion syndrome), a specific combined immune deficiency which occurs in 1 in 4,000 births (5) has common comorbidities of autism spectrum disorder and attention deficit hyperactivity disorder (6). Learning disabilities in DiGeorge syndrome are thought to be caused by haploinsufficiency of one or multiple genes located at the 22q11.2 region, resulting in a lack of specific proteins impacting stages of neurodevelopment (7).

The process of transition from paediatric to adult healthcare involves a complex series of changes in psychosocial and environmental development alongside the biological changes associated with puberty (8). Patients with learning disabilities are often well supported during childhood years with systems in place to support their development and needs whilst in the schooling system. This can be more difficult and complicated once they have grown out of this, which often corresponds with transitions in medical care. For those with chronic health problems such as an immunodeficiency and learning disabilities, there are additional challenges that occur during healthcare transition. These may include managing care from multiple different health and social care teams, whilst also navigating the varying philosophies and practises that different healthcare services use (9). This process occurs at a turbulent time where patients may be struggling with a multitude of psychosocial changes in their lives in addition to keeping on top of their complex health needs, which they may find difficult to cope with (9). Research has suggested that individuals with learning disabilities and their families are at higher risk for developing mental health difficulties during the transition process (10) and the transition process itself may have a negative influence on general health and wellbeing (11). In order to increase the chances of a successful transition, there are a number of recommendations that can be summarised from the literature.

## Interactions with healthcare professionals

Supportive and meaningful interactions with healthcare professionals can help to increase the quality of the transition experience. A UK hospital survey found that only 23% of healthcare professionals asked the young person with a learning disability if they would prefer to be seen alone, with only 43% of doctors thinking it was an essential question to ask (12). Research has also found that young adults with complex health problems and learning disabilities, who have been in the healthcare system for a prolonged period, can develop significant relationships with their paediatrician, which when lost can cause some distress (11). At times, it was reported that doctors may often resist discharging young adults with learning disabilities which discouraged the adolescents from starting to use adult healthcare services and increase their independence, and that case managers were more likely to treat young adults with independence and autonomy which supported this change (11).

Terminology used during appointments may also need to be reviewed as young adults with learning disabilities may not be able to comprehend the metaphors that doctors frequently use and explanations of anatomy or procedures may need to be simplified (13). These factors are especially important in transitional care as clear communication can be a predictor of successful transition (13). Transition nurses can act as a link between families and doctors and help to increase feelings of continuity (8) by providing emotional support, guiding the adolescent and their family through the transition process and helping with the planning of care (8).

The Department of Health has made efforts to emphasise that healthcare services need to play a significant role in ensuring that people with learning disabilities’ needs are met in policy (14), Training is emphasised in policy as the Department of Health’s ‘Better services’ highlights the significance of staff training for those working with people with learning disabilities and emphasises a person-centred approach to care (15). However, research has shown that in the UK, clinicians and administrative staff did not have a good understanding of autism and other associated learning disabilities, despite working in a healthcare service for those with learning disabilities (16). Further work is required to address this.

## Tools to aid transition

There are also different tools and documents that can assist the transition process. Research in Canada has suggested the use of a ‘Health Passport’ to be helpful in ensuring that doctors quickly gain access to vital medical information (16). In Ireland, researchers produced a tool in collaboration with participants who had DiGeorge syndrome, to help increase the quality of communication between doctors and patients (17). This tool enables clinicians to see an overview of the patient’s symptoms and comorbidities, with space to make notes regarding their consultation and a monthly calendar is included to help the patient manage their large number of appointments (17).

Research has suggested that government and privately funded disability service case managers were able to assist families that were struggling (11) by specifically communicating between services for families, organising appointments and ensuring that the families felt supported during this time (11). Young people with learning disabilities may prefer to make decisions in collaboration with their family, rather than independently, and feelings of inadequacy could arise when pushed to act independently (18). On the other hand, paternalistic relationships may arise between paediatricians and patients because of the close nature of the care of the young adult (19). Although this type of relationship serves to protect the care of the adolescent, it needs to be acknowledged alongside the young person’s need for autonomy (19).

## Person-centred care

In order to tackle these issues of autonomy and independence, family and person-centred care can be prioritised by making the individual and their families the main focus of care. A person-centred transition plan draws together all of the young adult’s existing care plans and includes information on how the adolescent can best stay healthy, information on how to communicate best with the adolescent, and also information on the patient’s likes, dislikes and plans for the future (20). Often a Circle of Support is included within this pathway, which involves regular meetings of people important to the young adult’s care such as family, friends and healthcare professionals, and where their goals in life can be discussed and plans can be actualised to ensure that there is also someone to support the young person (20). Person-centred transition pathways are key to ensuring that the individual is involved in the planning of their care, and it takes into consideration contextual information, future plans and the patient’s general life circumstances (12). Personalised transition models involving education and skills training are important and must be flexible to adapt to the changing needs of the adolescent as they mature and also the ever-changing process of transition itself (18). Importantly, discussions around consent of care and privacy should also be vital parts of this pathway when the individual turns 16 alongside continually assessing transition readiness from around the start of the young adult’s teenage years (13).

Often the transitional period and decision making can be difficult for family and clinicians to adjust to. Poor understanding of the Mental Capacity Act (21, 22) can cause uncertainty in situations where the young adult lacks the capacity to make specific healthcare decisions. The decision maker more often switches from parent to clinician in cases of adults who lack capacity, and this can invoke a sense of loss of power or control in the parent/carer. It can also lead to delays in treatment when clinicians are unsure about the consent and best interest process, in particular when a young person is in care. Discussions and reassurance around how these decisions will be made are important in helping the young person and family navigate the transitional process.

## Support services

Support services such as advocacy groups, mentoring and psychological support can increase confidence and help manage expectations for the future (10, 11, 21, 23, 24). Family counselling and advocacy groups in particular, can be beneficial for families of adolescents with comorbidities of mental illness (10, 13) and can be used to empower the adolescent in their decision-making processes (11), the same could be suggested for patients with learning disabilities. These services allow for parents or carers to understand the adult healthcare system and find strategies to increase their young person’s levels of independence (13). This highlights not only the importance of independence, that should be acknowledged in adolescents with learning disabilities, but also that there are support services available to help those with chronic health conditions specifically. Because some of these services are offered to families and carers, as well as the individuals with learning disabilities (10) the adolescents can seek support outside of a medical setting.

## Discussion

This paper aimed to address some recommendations from the research on supporting transition for adolescents with immunodeficiency and learning disabilities. Due to the lack of research available on young adults with learning disabilities and immunodeficiency, the recommendations in this article have been gathered from research conducted on adolescent populations who suffer from other chronic health conditions, with similar symptom phenotypes to immunodeficiency, to develop a more accommodating and understanding process for those with learning disabilities and an immunodeficiency.

From the research, one of the most important recommendations from this review is that considerations need to be made by those interacting with young adults with learning disabilities. Multiple research papers have emphasised the importance of supportive interactions from clinicians (8, 11, 12) and family members (20) so that those with a learning disability and chronic health condition, such as an immunodeficiency, can have a transition process that accommodates their specific needs. In order for this to occur, further awareness and training is needed for medical professionals working with these individuals and psychological interventions such as family counselling (10, 13) can help increase the adolescent’s wider support network. This may involve including the young adult in decision-making process, to the extent that their learning or intellectual disability allows, in order for the transition process to feel as autonomous as possible.

Ensuring continuity of healthcare is also an important recommendation for those with learning disabilities (16). Healthcare transition occurs at a difficult time in any young person’s life, regardless of whether they have learning difficulties or not and maintaining contact with both paediatric and adult doctors through a transition nurse or case manager can maintain feelings of continuity (8, 11) throughout the transition process. Alongside transition nurses and case managers, person-centred care should be incorporated into transition to increase feelings of continuity and autonomy. It is also important to note, that young people, both with and without a comorbid learning disability, experience a range of issues (e.g., mental health, sexual wellbeing, life expectancy) that arise as the individual goes through adolescence (9). These issues need to be considered for all transition patients, and appropriate adaptions for patients with comorbid learning disabilities, in order for a clinician to treat the young person holistically.

Parents of adolescents with learning disabilities and an immunodeficiency should be supported throughout the transition process with psychological interventions. Research on those with special health care needs and disabilities has suggested that family counselling can be beneficial (10, 13) in educating families about the transition process and specific techniques for supporting their child’s independence (13).

## Data availability statement

The original contributions presented in the study are included in the article/**Supplementary Material**. Further inquiries can be directed to the corresponding author.

## Ethics statement

Ethical approval was not required for the study involving humans in accordance with the local legislation and institutional requirements. Written informed consent to participate in this study was not required from the participants or the participants’ legal guardians/next of kin in accordance with the national legislation and the institutional requirements.

## Author contributions

EA and MC contributed to conception of the article. EA wrote the first draft of the manuscript. MC and SL wrote sections of the manuscript. All authors contributed to manuscript revision, read, and approved the submitted version.
