# Supplementary material for: Transition for adolescents with learning disabilities and an immunodeficiency
Source: Front Immunol. 2023 Sep 13;14:1211872. doi: 10.3389/fimmu.2023.1211872 (PMC10533907; doi:10.3389/fimmu.2023.1211872)
Supplement: Supplementary file 1 [file DataSheet_1.pdf]

TRANSITION FOR ADOLESCENTS WITH LEARNING DISABILITIES AND AN IMMUNODEFICIENCY

Background

Research has shown that different types of immunodeficiencies, such as combined immunodeficiencies, may present with higher rates of comorbid learning or intellectual disabilities.

Patients with intellectual disability are well supported during childhood years with systems in place to support their needs, but this can be more difficult and complicated once they move to adult services.

19% of patients with severe congenital intellectual disability are also diagnosed with a **learning disability**

Individuals and their families are at higher risk for mental health difficulties during the transition process.

The healthcare transition process itself can have a negative influence on general health and wellbeing.

Interactions with healthcare professionals

- Supportive and meaningful interactions with healthcare professionals can help to increase the quality of the transition experience.
- Young adults with complex health problems and learning disabilities can develop significant relationships with their paediatrician, which when lost can cause distress.
- Clear communication can be a predictor of a successful transition. The terminology used during appointments may also need to be considered.
- Transition nurses can act as a link between families and doctors and help to increase feelings of continuity.

Tools to aid transition

- Research suggests the use of a 'Health Passport' which can be helpful for doctors to quickly gain access to vital medical information.
- Disability service case managers were able to assist families that were struggling with the transition process.

Support services

- Support services such as advocacy groups, mentoring, and psychological support can increase confidence and help manage expectations for the future.
- Family counselling and advocacy groups, in particular, can be beneficial for families of adolescents with comorbidities of mental illness and can be used to empower the adolescent in their decision-making processes.

Person centred care

- Person-centred care can be prioritised by making the individual and their families the main focus of care.
- A person-centred transition plan draws together all of the young adult's existing care plans and is key to ensuring that the individual is involved in the planning of their care.
- Personalised transition models involving education and skills training are important and must be flexible to adapt to the changing needs of the adolescent as they mature.
- Poor understanding of the Mental Capacity Act (2005) can cause uncertainty in situations where the young adult lacks the capacity to make specific healthcare decisions. Discussions and reassurance around how these decisions will be made are important in helping the young person and family navigate the transitional process.
